# Streptococcus gallolyticus Infection Associated With Colorectal Adenoma in a Patient With Type 1 Diabetes Mellitus: A Case Report

**DOI:** 10.7759/cureus.88257

**Published:** 2025-07-18

**Authors:** Hayato Isobe, Takatoshi Anno, Fumiko Kawasaki, Kohei Kaku, Hideaki Kaneto

**Affiliations:** 1 Department of General Internal Medicine 1, Kawasaki Medical School, Okayama, JPN; 2 Department of Diabetic Medicine, Kurashiki Central Hospital, Kurashiki, JPN; 3 Department of Diabetes, Endocrinology and Metabolism, Kawasaki Medical School, Okayama, JPN

**Keywords:** colorectal adenoma, glycemic control, sepsis, streptococcus gallolyticus infection, type 1 diabetes mellitus

## Abstract

We present a case of *Streptococcus gallolyticus* sepsis in a patient with type 1 diabetes mellitus (T1DM) who had poor glycemic control. Once the sepsis and infection had improved, we examined the patient for colorectal adenoma or carcinoma and detected a stalked polyp with colonoscopy. Therefore, this case underscores the importance of actively screening for malignant colonic diseases in patients with *S. gallolyticus* infection. We believe that clinicians should be aware of this association as it demonstrates how to perform gastroenterological follow-up for patients infected with *S. gallolyticus*, particularly those with poorly controlled DM.

## Introduction

*Streptococcus bovis* is a common commensal bacterium found in the intestinal tract of healthy patients. Recently, *S. gallolyticus* was distinguished from *S. bovis* species due to its distinct associations with gastrointestinal pathology, and *S. gallolyticus* has been clinically linked to colonic malignant diseases [1,2]. A large seroepidemiological study reported a significant association between exposure to *S. gallolyticus* antigens and the risk of colorectal adenoma or carcinoma [3].

Patients with diabetes mellitus (DM) are immunocompromised hosts; therefore, they may develop inflammatory diseases, especially when glycemic control is poor for an extended period. Additionally, it has been reported that patients with DM have a high frequency of various malignant diseases, and thus, it is important to pay attention to the onset of various malignant diseases.

In this report, we present a case of type 1 DM (T1DM) with colorectal adenoma, which was diagnosed under *S. gallolyticus* sepsis conditions. We believe that it is important for clinicians to know this subject because it shows how to perform gastroenterological follow-ups for patients with T1DM under *S. gallolyticus* infection conditions.

## Case presentation

A 53-year-old Japanese man with T1DM was referred to our hospital because of an infection. He was diagnosed with T1DM, hypertension, and dyslipidemia at the age of 39, and he underwent a mitral annuloplasty for mitral regurgitation at the age of 50. He was treated with insulin, which included insulin lispro (15-13-19 units/day) and insulin degludec (16-0-20 units/day). His height, body weight, and body mass index were 151.2 cm, 54.8 kg, and 24 kg/m^2^, respectively. His vital signs were as follows: temperature, 37.9°C; blood pressure, 114/67 mmHg; heart rate, 101 beats per minute; and oxygen saturation, 95 % (room air). Table 1 lists the laboratory data from the emergency room. Diabetes-associated data were as follows: plasma glucose, 360 mg/dL; glycated hemoglobin, 9.6%; and anti-glutamic acid decarboxylase antibody, 70.3 U/mL. Ketone body levels were slightly elevated. Inflammation-associated data drastically increased: white blood cell count, 20,830/μL (neutrophils, 94.8%); C-reactive protein, 37.04 mg/dL; and procalcitonin, 6.88 ng/mL. Thus, we initiated whole-body management for infections and sick days with diabetes.

**Table 1:** Laboratory data of the patient in the emergency room AST: aspartate aminotransferase, ALT: alanine aminotransferase, ALP: alkaline phosphatase, LDH: lactate dehydrogenase, γ-GTP: gamma-glutamyl transferase, BUN: blood urea nitrogen, GAD Ab: glutamic acid decarboxylase antibodies, CRP: C-reactive protein, PCO_2_: partial pressure of carbon dioxide, PO_2_: partial pressure of oxygen, HCO_3_-: bicarbonate, BE: base excess, SO_2_: oxygen saturation

Variable	Result	Reference range
Peripheral blood		
White blood cells	20,830/μL	3,300-8,600/μL
Neutrophil	94.8%	52%-80%
Red blood cells	388×10^4^/μL	386-492×10^4^/μL
Hemoglobin	12 g/dL	11.6-14.8 g/dL
Hematocrit	34.6%	35.1%-44.4%
Platelets	34.7×10^4^/μL	15.8-34.8×10^4^/μL
Blood biochemistry		
Total protein	5.3 g/dL	6.6-8.1 g/dL
Albumin	2.1 g/dL	4.1-5.1 g/dL
Globulin	3.2 g/dL	2.2-3.4 g/dL
Total bilirubin	0.5 mg/dL	0.4-1.5 mg/dL
AST	25 U/L	13-30 U/L
ALT	13 U/L	7-23 U/L
LDH	364 U/L	124-222 U/L
ALP	115 U/L	106-322 U/L
γ-GTP	54 U/L	9-32 U/L
BUN	33 mg/dL	8-20 mg/dL
Creatinine	2.12 mg/dL	0.46-0.79 mg/dL
Cholinesterase	225 U/L	201-421 U/L
Uric acid	5.6 mg/dL	2.6-5.5 mg/dL
Total cholesterol	131 mg/dL	142-248 mg/dL
Sodium	124 mmol/L	138-145 mmol/L
Potassium	4.2 mmol/L	3.6-4.8 mmol/L
Chloride	93 mmol/L	101-108 mmol/L
Diabetes marker		
Plasma glucose	360 mg/dL	-
Hemoglobin A1c	9.6%	4.9%-6.0%
Total ketone body	272.9 μmol/L	0-130 μmol/L
Acetoacetate	121.6 μmol/L	0-55 μmol/L
β-Hydroxybutyrate	151.3 μmol/L	0-85 μmol/L
GAD Ab	70.3 U/mL	<5 U/mL
Infectious marker		
CRP	37.04 mg/dL	<0.14 mg/dL
Procalcitonin	6.88 ng/mL	0.00-0.05 ng/mL
Blood gas analysis		
pH	7.434	7.360-7.460
PCO_2_	33.6 mmHg	34-46 mmHg
PO_2_	71.8 mmHg	80-90 mmHg
HCO_3_^-^	22.1 mEq/L	24-32 mEq/L
BE	-1.1 mEq/L	-2.5-2.5 mEq/L
SO_2_	93.9%	95%-98%
Urinary test		
Urinary pH	6	5.0-7.5
Urinary protein	2+	-
Urinary sugar	3+	-
Urinary ketone body	-	-
Urinary bilirubin	-	-
Urinary blood	2+	-
Urinary bacteria	-	-

We started antibiotic therapy for the infection (3 g/day of meropenem) and examined the source of the infection. Three days later, *S. gallolyticus*, the pathogenic bacteria, were detected in the blood. Since it is known that *S. gallolyticus* infection is associated with infective endocarditis and pyogenic spondylitis, we performed an echocardiographic examination and abdominal computed tomography scan immediately after the detection of *S. gallolyticus*. We did not detect infective endocarditis, although the patient had pyogenic spondylitis. Subsequently, we added vancomycin (2 g/day) and changed the antibiotics based on their sensitivity to *S. gallolyticus*. Finally, the infection improved, and the antibiotics were discontinued.

**Figure 1:**
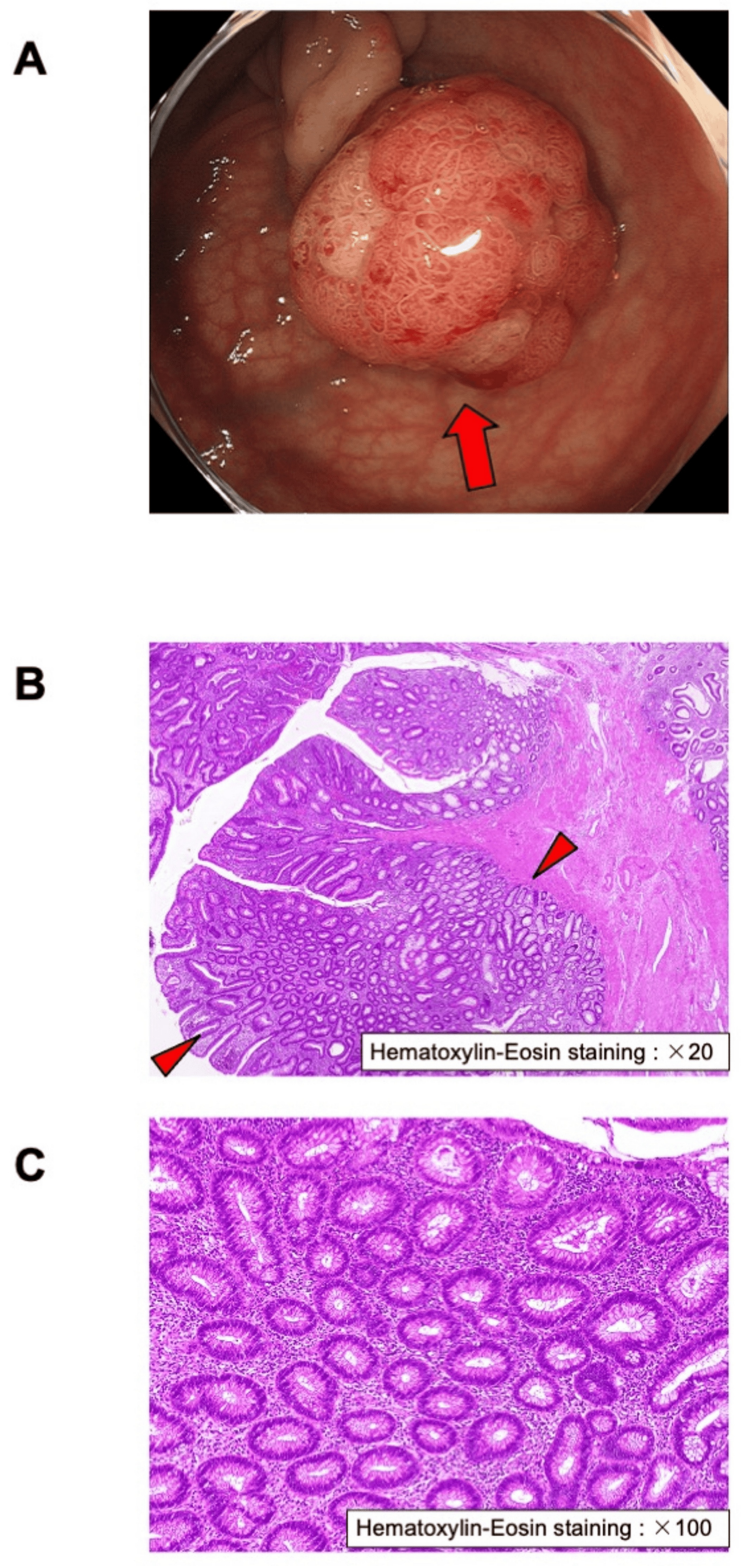
Colorectal adenoma polyp in this patient A: Colorectal adenoma polyp on colonoscopy. A stalked polyp (20 mm in diameter) was detected (red arrow). B (hematoxylin and eosin staining, ×20) and C (hematoxylin and eosin staining, ×100): Histopathological microscopic findings. Colorectal tubular adenoma (red arrowhead) was observed together with large round nuclei and dysplasia.

When *S. gallolyticus* infection is observed, especially in sepsis, we should be aware of the possibility of colorectal cancer. Therefore, we examined whether this patient had colon disease. His fecal occult blood test result was positive twice. The tumor markers carcinoembryonic antigen and carbohydrate antigen 19-9 were 5.1 ng/mL and 17.2 U/mL, respectively. Furthermore, we performed a colonoscopy after the patient’s symptoms improved. A stalked polyp (diameter: 20 mm) was detected and resected. As shown in Figure 1, we diagnosed this patient with a pathological colorectal adenoma.

## Discussion

There is a complex ecosystem in the colon, which is characterized by the presence of rich and diverse microbiota. Although the complex bacterial community population is fairly stable under healthy conditions, intestinal diseases disturb the ecological balance and alter the intestinal microbiota. It has been established that an over-representation of specific bacterial species in the microbiota is closely associated with colorectal cancer [4,5]. *Streptococcus bovis* is a common commensal bacterium present in the intestinal tract of healthy patients [6]. Since it seems that each *S. bovis* subspecies has different associations with gastrointestinal pathology, it has recently been considered that *S. bovis* is not present as a single bacterial entity. Among them, *S. gallolyticus* is one of the pathogens associated with colorectal cancer [1,2]. The prevalence of *S. gallolyticus* infection was reported to be about 3.2% in an unselected cohort of patients with colorectal cancer [7].

It is controversial whether the presence of colorectal cancer makes patients more susceptible to *S. gallolyticus* infection. Direct epithelial or metabolic changes, both of which are induced together with the development of colorectal cancer, bring about a competitive advantage for intestinal bacteria. Besides a possible causative association between the intestinal microbiota and colorectal cancer, the progression of colorectal cancer per se may affect the local microbial population [8].

The physiological changes that are accompanied by the development of colorectal cancer include an increased infiltration of intestinal bacteria. The presence of a tumor has been reported to be beneficial for some opportunistic intestinal pathogens [8]. Therefore, the increased infiltration of *S. gallolyticus* is possibly responsible for endocarditis and bacteremia. It was reported that bacteremia caused by *S. gallolyticus* was significantly correlated with colorectal adenoma and carcinomas [1] and that blood invasion occurred through bacterial translocation, but not through the formation of ulceration of the mucosa [9]. Also, it is considered that sites of neoplastic lesions can accelerate the passage of *S. gallolyticus* into the blood flow [10].

In contrast, there are some reports that the amount of *S. gallolyticus* is approximately 10-fold higher in neoplastic colonic tissues than in healthy conditions [5]. Also, it has been reported that advanced adenomas and invasive carcinomas are the main causes of this increased neoplasia caused by *S. gallolyticus* [11]. Colonization of *S. gallolyticus* is present in cancerous tissue, and colorectal cancer-specific conditions lead to gut colonization of *S. gallolyticus* [5,12]. Based on these findings, *S. gallolyticus* may be favorably associated with advanced adenomas. Another possibility is that adenomas occur at an advanced stage when *S. gallolyticus* is present.

It is important to note that some patients with colorectal adenoma and carcinoma have no gastrointestinal clinical signs or symptoms [6]. *Streptococcus gallolyticus* infection in patients with colorectal cancer suggests that these infections can occur quite commonly without symptoms. Also, it was reported that *S. gallolyticus* was present in the blood of a healthy blood donor who was later diagnosed with colon cancer [13]. Therefore, when *S. gallolyticus* is detected in patient blood cultures, we should consider performing a colonoscopy.

Interestingly, in the human intestine, *S. gallolyticus* is one of the predominant species of bacteria in the ileum [14] and plays an important role in carbohydrate consumption [15]. It is considered that the nutritional status of colorectal cancer tissue is quite similar to that of the ileum, with high concentrations of lactate, carbohydrates, and their derivatives [15,16]. Since it has been suggested that the intestinal epithelial barrier is disrupted in animal models with T1DM [17], that may be true for patients with T1DM as well. Patients with T1DM have many complications due to chronic hyperglycemia, increased inflammation, and immune abnormalities. Therefore, patients with T1DM are more susceptible to infection, especially when glycemic control is poor. Moreover, DM itself is thought to increase the risk of colorectal cancer [18]. In this case, *S. gallolyticus* sepsis, colorectal adenoma, and T1DM may be closely related to each other. Therefore, in the case of *S. gallolyticus* sepsis, it is necessary to perform a careful assessment of possible colorectal cancer and a routine endoscopic follow-up, even when colorectal adenoma and carcinoma are not identified. Needless to say, another study with a larger sample size and functional experiments needs to be conducted to verify the association of *S. gallolyticus* infection and T1DM, and the mechanism under these phenomena merits further investigation. However, diabetic patients with poor glycemic control may develop sepsis and require treatment, and blood culture testing may be performed. Therefore, what we can conclude at present is that clinicians should keep in mind the importance of gastroenterological follow-ups after the detection of *S. gallolyticus*.

## Conclusions

Since patients with poorly controlled DM are immunocompromised hosts, they are often tested with a blood culture test for infection. It is important to check malignant colonic diseases more actively and to regularly perform gastroenterological follow-ups if *S. gallolyticus* is detected in infection or sepsis. More than anything, even clinicians should be aware that *S. gallolyticus* infection is closely associated with malignant colonic diseases. Above all, good glycemic control may lead to the prevention of a variety of diseases, including rare bacterial infections, such as *S. gallolyticus* infection.
